# Endoparasitic Diseases in Breeding Kennels: A Frequent and Complex Problem Requiring a Holistic Approach

**DOI:** 10.3390/ani14162357

**Published:** 2024-08-15

**Authors:** Aurélien Grellet, Hanna Mila

**Affiliations:** NeoCare, Reproduction, ENVT, Université de Toulouse, 31300 Toulouse, France; aurelien.grellet@envt.fr

**Keywords:** dog, breeding, kennel, canine parasite, abortion, neonatal mortality, weaning diarrhea

## Abstract

**Simple Summary:**

All over the world, kennels are held by different kinds of breeders: occasional, regular hobby, and professional breeders. Even if these dog breeders have various breeding practices, manage canine populations of very different sizes, and have various final purposes (i.e., companion dogs or working dogs), all conscientious breeders of dogs have the same priority: the health and wellbeing of their animals. Although parasites are extremely common pathogens in all dogs, they are particularly problematic in breeding kennels as they can interfere with breeding performance and the health of dogs. This review aims to explain why breeding kennels are predisposed to parasitic infestations, how the most common parasites can influence dog breeding performance and health, and how to prevent parasite infestations in this specific environment.

**Abstract:**

Parasitic infestations in dogs are frequent, particularly in breeding kennels, being a cause of suffering in animals and economic loss for breeders. In breeding bitches, some parasites may cause abortion, and in puppies they may be responsible for neonatal mortality, weaning diarrhea, or neurological clinical signs. This review aims to investigate the factors of predisposition, diagnostics, and control in relation to the most frequent parasitic diseases in breeding kennels. It highlights that the control of parasitic diseases in dogs at the population level is complex. A holistic multidisciplinary and pluritechnical approach is thus needed to deal with endoparasitoses.

## 1. Introduction

The number of companion animals, such as dogs, is increasing worldwide each year. According to a report of the trade body representing European pet food—FEDIAF (Fédération Européenne de l’Industrie des aliments pour Animaux Familiers)—in 2022, 25% of all European households owned at least one dog [1]. Although the origins of these animals may differ (e.g., private parties, animal shelters, or dog breeders), the breeding of bitches is necessary to obtain puppies.

According to a French study on 45,913 mated bitches and their 204,537 puppies, pregnancy failure in bitches is 12% on average, and the mortality rate in puppies between birth and two months of age (age at sale) is estimated at 13% [2]. Other studies present morbidity rates in puppies from breeding kennels at about 35% [3], with digestive disorders being the most often cause of morbidity at weaning (25% of puppies present with diarrhea between 6 and 8 weeks of life) [4]. Moreover, reproductive performance in canine species varies among breeds, among kennels, and even among dogs from the same kennel.

The origin of reproductive troubles in bitches and morbidity and mortality in puppies is varied. These troubles can be alimentary, environmental, linked to the management of the dogs, or infectious, with one cause of infection being parasites. In one study on 266 puppies from 29 kennels, 77% of puppies presentedat least one parasite in their feces, showing that parasitic infestation is a widespread problem in canine facilities [4]. Some endoparasites may be responsible for abortion in breeding females, others for stillbirth or neonatal mortality, and others for neurological or digestive disorders. The most common parasites found in dogs housed in breeding kennels, as well as the clinical signs presented in infested dogs, are mentioned in Table 1.

As parasitic infestation may be an origin of suffering for animals and also cause financial trouble for breeders, this review aims to investigate the factors of predisposition, diagnostics, and control in relation to parasitic diseases in breeding kennels.

## 2. Dog Breeding Kennels: Favorable Environments for Parasitic Infestation

The prevalence of parasites varies from one study to another. It is influenced by geography, detection methods, whether a dog is symptomatic or not, deworming protocols, the age of the animals, their lifestyle (i.e., hunting), and housing conditions [21]. Dogs living in breeding kennels or in shelters are more frequently infested (or infected in the case of protozoa) by intestinal parasites (*Toxocara canis*, *Giardia* sp., *Cystoisospora* spp.) than household dogs. For example, the prevalence of *Giardia *sp. in dogs living in breeding kennels or shelters is twice as high as in individually housed dogs [22]. Moreover, this prevalence increases with the size of the kennel. For instance, the prevalence of *Giardia *sp. is 3.5 times higher in kennels producing more than 30 puppies per year than in kennels producing less than 30 puppies per year (63.2% vs. 17.7%) ([4] and authors’ unpublished data). This higher prevalence can be explained by several factors that can have an influence alone, or be associated: the life cycle of parasites, their contagiousness, their stability in the environment, the presence of susceptible animals with low immunity, the density of animals, and the hygiene protocols applied in kennels.

Some parasites can take advantage of the reproduction cycle of their host to infest new generations. For example, dog fetuses can be infected in utero by *T. canis* somatic larvae from day 42 of gestation (the most significant mode of transmission in dogs) [23]. Factors inducing this reactivation of somatic larvae and intrauterine infestation are still unknown. A modification of the hormonal status of pregnant bitches is suspected. Activated somatic *Toxocara* larvae can also be transmitted to neonates via colostrum and milk. Transplacental transmission is also a route of infection of other parasites like *Neospora caninum*, *Toxoplasma gondii*, *Leishmania infantum*, and *Anaplasma platys* (Table 2).

Moreover, the majority of parasites circulating in breeding kennels are particularly resistant in this environment (Table 3). *Giardia* sp. can survive for several months outside a host in wet and cold conditions (+4 °C). This environmental resistance is even more important in other intestinal parasites like *T. canis*, whose eggs can survive under optimal circumstances in the soil for at least one year. The age of dogs also influences the risk of infestation, which is higher in very young puppies [5]. Indeed, a retrospective study on 3590 fecal samples demonstrated that most *Cystoisospora* infections (78%) were found within the first 4 months of life, whereas dogs older than 1 year rarely (1%) shed oocysts [35]. Such an effect of age on parasitic infestation could be explained by the need to develop an acquired immunity during the growing period, as suggested by Urquhart (1990) [36].

## 3. Clinical Forms of Parasitosis in Breeding Kennels

### 3.1. Resorption and Abortion

The actual risk of embryo or fetal resorption and abortion due to parasite infestation seems very low. Even though the transplacental transmission of *N. caninum* has been observed in dogs, only three studies have evaluated the consequences of experimental infection with this parasite in pregnant bitches [29,30,38]. A total of 13 bitches were inoculated. Five out of six bitches presented resorption or macerated fetuses in one of these studies [30], whereas no effect was observed during gestation in the other two studies [29,38]. The variability in the clinical signs observed can be linked to the strain of *Neospora*, the method of inoculation, the number of tachyzoites administered, and the time of inoculation during gestation. Among the 34 puppies born alive in these three studies, the mortality rate during the first 21 days of age was 56%. However, the limitation of these studies was the lack of control groups (non-infested). A recent epidemiological study failed to demonstrate any relationship between *N. caninum* seropositivity and reproductive disorders in dogs [39]. Toxoplasmosis as a primary disease is extremely rare in dogs, and is mostly observed in cases of immunosuppression [17]. However, some sporadic cases of abortion have been demonstrated in bitches after oral primo-infection during gestation. Indeed, signs of placentitis and fetal infection were observed in the aborted fetuses, with *Toxoplasma gondii* isolated in their internal organs [18,32]. Even if a high frequency of transplacental transmission of Leishmania infantum was observed in puppies (32% of fetuses infected in naturally infected bitches), abortion due to this parasite in dogs seems rare. Indeed, only one case of Leishmania-associated placentitis and abortion has been described in a dog [26].

### 3.2. Neonatal Mortality

As mentioned above, some parasites may be responsible for increased mortality during the first 21 days after birth (defined as the neonatal period). *Toxoplasma gondii* and *Neospora caninum* were observed in 41 stillborn puppies from 23 litters, suggesting that these parasites may be responsible for stillbirth in dogs [27]. Toxocariasis in newborns may also be lethal, as larvae migration in infested puppies during the first weeks after birth may contribute to tissue damage or bacterial translocation from the gut to the internal organs [40]. *Ancylostoma* infestation via colostrum was associated with severe anemia and death in puppies between two and three weeks of age [41]. Some sporadic cases of neonatal mortality in puppies infected with *Cryptosporidium* sp., *Coccidia* sp., or *Encephalitozoon cuniculi* are reported, although very few data are available on this subject.

### 3.3. Neurological Signs in Puppies

Some parasitic infections, i.e., *Neospora caninum*, *Toxoplasma gondii*, and *Encephalitozoon cuniculi* infection, can induce neurological signs in puppies. *Neospora caninum* can induce ataxia and hind limb paresis, which develops into progressive ascending paralysis in congenitally infected puppies. Most infected dogs are born asymptomatic and begin to develop clinical signs three or more weeks after birth. In the same litter, only some puppies may be congenitally infected, and some may develop clinical signs. Four different studies evaluated serological status and clinical signs in a total of 156 puppies born from serologically positive bitches [28,42,43,44]. In these studies, 7.3% (10/137) of puppies were found seropositive during their first weeks/months of age, and 6.4% (10/156) developed clinical signs of neosporosis. Even if congenital infections were suspected in these studies, postnatal infection cannot be ruled out, as the majority of serology tests were performed several weeks after birth. The seroprevalence of *Neospora caninum* seems low in adult breeding bitches, with 7.3% of dams seropositive. Toxoplasma gondii, an intracellular coccidian parasite, can induce clinical signs similar to *Neospora caninum* (seizures, ataxia, and paresis or paralysis). In young dogs under 1 year of age, generalized toxoplasmosis can be observed with icterus, fever, dyspnea, and diarrhea. A third intracellular parasite, *Encephalitozoon cuniculi*, can induce neurologic problems in puppies between 4 and 10 weeks of age. Puppies show signs of renal failure and neurologic signs such as depression, ataxia, blindness, and convulsions [45].

### 3.4. Digestive Disorders before Weaning

Different intestinal parasites, with *Giardia* sp., *T. canis*, and *Isospora* sp. isolated most often, can induce digestive disorders in puppies (Table 1). However, their pathogenicity depends on age, the immunity of puppies, and coinfection by other enteropathogens. For example, *Cystoisospora ohiensis* (coccidia) can cause enteric disorders in very young animals (as early as 7 days of age) but does not affect puppies at weaning, whereas *C. canis* mainly induces clinical signs in puppies at weaning and, more particularly, after stress. However, the pathogenic power of one given parasite is difficult to evaluate per se, since several parasites can be simultaneously present within the digestive tract. A study on 316 puppies revealed that 40% of them excreted at least two different intestinal parasites, underlying the necessity for a holistic evaluation of parasites excreted [4]. In addition to parasites, a strong impact of canine parvovirus on weaning diarrhea was observed in the cited study (61.5% of infected puppies presented abnormal feces compared to 15.2% of puppies not infected by this virus). Thus, a transdisciplinary approach associating parasitology and virology is mandatory for optimal diagnosis in puppies presenting weaning diarrhea. Some of the most ubiquitous intestinal parasites have been described in this study. However, veterinarians should be aware of the possible emergence of new parasites, such as *Pentatrichomonas hominis* and *Blastocystis hominis*, which have recently been isolated in diarrheic puppies living in breeding kennels and households [16,46,47].

## 4. Diagnosis of Parasitic Circulation in Kennels

When a group of dogs are experiencing parasitic health problems in a kennel, not all cases may be resolved with a single treatment, and it is necessary to target the contributory factors as well as the causative agents. To provide the breeder with adapted recommendations in terms of kennel management, the veterinarian would need to visit the kennel. Indeed, the veterinary visit allows an understanding of the breeding establishment as a whole. A particular attention should be paid to cleaning and disinfection procedures, the organization of the kennel, the animals’ housing, the management of the dogs, and feeding practices. This is also an opportunity to collect some biological samples (feces, blood, or parasites themselves) for complementary analyses. The diagnostic method to identify the parasites present in the kennel should be adapted depending on the diagnostic hypothesis, the available biological samples, the severity of the situation, and the necessity of quantifying parasitic load. To decrease the cost of analyses and to limit the number of false-negative results due to the intermittent excretion of some parasites, pooling stools from several (three to five) dogs in one sample is proposed for kennels with large numbers of dogs [48]. An evaluation of pooled fecal samples (for the presence of enteropathogens) can be performed in three different populations of dogs: bitches in anestrus and stud dogs, pregnant and nursing females, and puppies around weaning. When several litters of different ages are present simultaneously in the kennel, two distinct pooled fecal samples can be submitted for examination: one sample from puppies aged between 4 and 6 weeks and another sample from puppies aged between 6 and 9 weeks. The method of detection should be adapted to the targeted parasite, as regular coproscopy techniques would not be sensible or sensitive to all the above-mentioned parasites.

## 5. Management of Parasite Circulation in Kennels

Parasite prevention and control measures can be divided into two main categories: preventing the introduction of the parasite and limiting its transmission.

Quarantine plays an important role in preventing the introduction of (mainly digestive) parasites. All newly introduced dogs need to be housed in a specific place isolated from the other dogs for at least 5 and ideally 14 days before being introduced into the kennel. During this period, a parasitological exam can be performed on these dogs, and they should be dewormed and groomed. Even if a future reproductive bitch or sire does not originate from an at-risk environment, such as another kennel, the risk of introducing a parasite still exists with the arrival of a new animal. Indeed, 4.6% to up to 11% of household dogs shed *T. canis* eggs in their feces [21,49,50], and 9% of privately owned dogs presented eggs in their hair in one study [51]. Food is also a potential mode of parasite introduction into kennels. Indeed, up to one-third of breeders feed their dogs with raw diets or bones [52], and raw meat products can be potentially contaminated with parasites like *Giardia* spp., *Neospora* spp., *Toxoplasma* spp., *Echinococcus* spp., *Cryptosporidium* spp., *Toxocara* spp., and more. On the other hand, the prevalence of these parasites in raw diets seems low [53], and the meat is often frozen for preservation. Freezing meat for at least 7 days is indeed a good strategy to prevent parasite infestation [54], as negative temperatures (<−15 °C) result in non-viable cysts in parasites such as *N. caninum*, *T. gondii*, and *C. parvum* [55,56]. To decrease the risk of parasitic transmission, excretion, persistence in the environment, and exposition to parasites need to be limited. To decrease excretion, a deworming program adapted to parasites circulating in the kennel must be proposed. As *T. canis* presents a zoonotic risk and since it is considered that almost 100% of dogs have been in contact with this parasite, a systematic deworming program targeting this parasite is of primary importance. To prevent the transmission of *T. canis* to fetuses during gestation, the European Specialist Counsel for Companion Animal Parasites (ESCCAP) recommends treating pregnant females with macrocyclic lactones (ivermectin, milbemycin oxime, selamectin, moxidectin) on the 40th and 55th day of pregnancy, or fenbendazole daily from the 40th day of pregnancy until the 14th day postpartum [57]. In order to decrease the infestation level in problematic kennels, puppies need to be treated from the age of 2 weeks and then every 2 weeks until the age of 2 months with fenbendazole/febantel, pyrantel, flubendazole, or nitroscanate. Lactating bitches must be treated at the same time as their puppies. The other dogs in the kennel should be treated at least every quarter.

Environmental conditions have an impact on the survival of parasites. Surface porosity, environmental temperature, and the presence of organic material are three factors influencing the survival of parasites. This underlines the importance of adapted flooring in kennel premises, i.e., it should be made of a resistant material with low porosity. Regular feces collection in pens, i.e., once per day, is recommended to reduce the circulation of parasites in the environment, but also to limit their accumulation in the soil. To limit the persistence of parasites in kennels, the efficient cleaning and disinfection of kennel buildings are mandatory. It is especially important to keep in mind that cleaning and disinfection are two separate steps. Cleaning, the first step, consists of the removal of visible organic material with soap or detergent, whereas disinfection, the second step, requires the application of a chemical or mechanical procedure to kill the remaining microbes. Cleaning alone reduces about 90% of microbes present on surfaces; thus, it should be performed daily in nurseries and at least once per week in zones housing healthy adults. The disinfectant used and the frequency of its use should be adapted to the circulating parasites (Table 4). In the case of *Isospora* sp. circulation, disinfection with quaternary ammonium is recommended every 48 h in case of an outbreak of coccidiosis, whereas disinfection once per week is sufficient outside of epizootic periods [58]. While a 30s exposure to 1% sodium hypochlorite solution (bleach) renders *E. cuniculi* non-infectious [59], *T.canis* eggs may remain infective after a 120 min long bath in 5.25% bleach [60]. To date, no commercially available disinfectants have been demonstrated to be efficient against *T. canis* [61]. Other mechanic methods can be used to reduce the circulation of some parasites in kennels (Table 4), such as high pressure or steam, with satisfying results against some common protozoa (*Cryptosporidium* sp., *Giardia* sp., or *Isospora* sp.).

To limit exposition to parasites, kennels have to be organized into separate sectors based on the differing vulnerability of different animals, determined by their physiological status. The presence of the following areas is thus recommended in breeding facilities: a maternity area (for pregnant bitches and their puppies until the first month of life), a nursery (for puppies and their dams from the second month of life until adoption), an area for adult dogs, an infirmary (for sick dogs), and a quarantine area (for new arrivals). Specific cleaning equipment for each sector should be available. The design should be arranged so that the movement of the staff through the facility should proceed from the areas housing the animals most susceptible to disease (puppies) and/or the healthiest animals (healthy adult dogs) to those who are likely to be a source of infectious disease (quarantine, infirmary).

## 6. Conclusions

A breeding kennel needs to be considered as an ecosystem, with interactions between hosts (dogs at a certain age, with certain genetics, immunity, etc.) and pathogens (different parasites with different pathogenicity, of different strains, etc.) influenced by breeding kennel management (breeders) and environment (population density, stress, hygiene protocols, temperature/humidity, etc.). A holistic multidisciplinary (parasitology, virology, immunology, ambiance analysis, etc.) and pluritechnical (PCR, coproscopy, kennel data analysis) approach to the problem is essential to evaluate this ecosystem. This way of thinking may improve the management of parasitic and infectious diseases in breeding kennels and the interpretation of clinical studies.

From a scientific perspective, other parasites should be considered in kennels for their potential role in canine infertility and neonatal mortality. Indeed, some blood parasites, such as *Anaplasma platys* and *Hepatozoon canis*, are highly prevalent in some regions [64], with possible transplacental transmission recently demonstrated in dogs [25,65].

Future studies on combating parasitic infestations in breeding kennels should also focus on several emerging issues and address key challenges. One promising avenue is the development of novel antiparasitic drugs with new mechanisms of action to prevent the rise of drug-resistant parasites pointed out recently in dogs and cats [66]. Additionally, advancements in vaccine development are crucial, as they could provide long-term immunity and reduce reliance on chemical treatments.

However, prospective studies must also consider the challenges inherent in these innovations. The development of drug resistance remains a significant concern, underscoring the need for continuous research and new drug discovery. Sustainable and eco-friendly solutions are critical to minimize the environmental impact of chemical treatments and ensure long-term viability. Furthermore, implementing these advanced strategies in breeding kennels requires overcoming barriers such as limited resources, insufficient infrastructure, and the need for effective education and training programs.

## Figures and Tables

**Table 1:** Prevalence of the most common endoparasites in breeding kennels worldwide and their clinical manifestations.

	Parasite	Prevalence ^1^	Tropism	Clinical Signs of Parasitosis	References
Protozoa	*Cystoisospora ohioensis*/*C. canis*	1.2–26.3%	Large intestine	Mucoid diarrhea	[4,5,6,7,8]
	*Cryptosporidium parvum*	5–25%	Small intestine	Intermittent diarrhea	[6,9,10,11]
	*Giardia* sp.	16.1–44%	Small intestine	Diarrhea, steatorrhea	[4,5,7,8,9,11,12]
	*Leishmania infantum*	2.5–10.6%	Skin and viscera	Stillbirth, dermatitis, weight loss, renal failure	[13,14]
	*Neospora caninum*	0.3–2%	Various cells	Stillbirth, neurological signs	[6,15]
	*Pentatrichomonas hominis*	15.8% ^2^	Large intestine	Chronic diarrhea	[16]
	*Sarcocystis* spp.	2–32.2%	Small intestine	Diarrhea	[6,12]
	*Toxoplasma gondii*	3–19%	All types of cells except red blood cells	Sporadic cases of placentitis and fetal death	[17,18]
Nematodes	*Ancylostoma caninum*	0.2–37%	Small intestine	Cough, pneumonia, hemorrhagic diarrhea	[7,11]
	*Strongyloides stercoralis*	0.3–1.2%	Small intestine	Profuse diarrhea, coughing	[14]
	*Toxocara canis*	0.2–26.3%	Small intestine	Cough, pneumonia, diarrhea, failure to thrive	[4,5,6,7,8,12,19]
	*Toxascaris leonina*	0.9–2.5%	Small intestine	Diarrhea, vomiting	[6,7,20]
	*Trichuris vulpis*	2.1–7.2%	Large intestine	Hemorrhagic colitis, anemia	[6,7,20]
	*Uncinaria stenocephala*	8.1%	Small intestine	Cough, pneumonia, hemorrhagic diarrhea	[11]
Cestodes	*Dipylidium caninum*	0.6%	Small intestine	Emaciation, hypoglycemia, neurological signs, diarrhea, anal pruritus	[6]
	*Taenia* sp.	0.3%	Small intestine	Diarrhea, colic, anal pruritus	[6,12]

^1^ Data obtained from healthy and sick animals via PCR or coproscopy. ^2^ Data on puppies only.

**Table 2:** Perinatal contamination of some parasites in canine breeding kennels.

Parasite	Semen	Placenta	Milk	Oral	Skin	References
*Ancylostoma caninum*	?	+	+	+	+	[24]
*Leishmania infantum*	+	+	-	+	-	[25,26]
*Neospora caninum*	?	+	?	+	-	[27,28,29,30]
*Strongyloides stercoralis*	-	-	+	+	+	[31]
*Toxocara canis*	-	+	+	+	-	[23]
*Toxoplasma gondii*	+	+	+	+	-	[27,32]
*Uncinaria stenocephala*	?	-	+	+	+	[33,34]

**Table 3:** Resistance of parasites in the environment [34,37].

	Moderate Resistance (Several Weeks)	High Resistance (Several Months)	Very High Resistance (Several Years)
Nematodes	*Strongyloides stercoralis*	*Ancylostoma caninum* *Uncinaria stenocephala*	*Toxocara canis* *Toxascaris leonina* *Trichuris vulpis*
Cestodes		*Dipylidium caninum * *Taenia* sp.	
Protozoa	*Encephalitozoon cuniculi*	*Cryptosporidium parvum * *Giardia duodenalis*	*Isospora canis* *Isospora ohiensis* *Sarcocystis* sp. *Toxoplasma gondii*

**Table 4:** Disinfection methods against parasites in kennels and their spectrum of action [62,63].

Method of sanitation		Spectrum of Action
Mechanic	High pressure (120–180 bar)	*Ancylostoma*, *Giardia*, *Neospora*, *Cryptosporidium*,
	Steam (temperature > 70 °C)	*Ancylostoma*, *Cystoisospora*, *Cryptosporidium*, *Giardia*
	Low temperature (−20 °C)	*Ancylostoma*, *Cryptosporidium*, *Giardia*, *Toxoplasma*
	UV light (0.2 mW/cm^2^)	*Ancylostoma*, *Cryptosporidium*, *Giardia*
Chemical	Alcohol	*Ancylostoma*, *Encephalitozoon*
	Ammonia	*Cystoisospora*, *Cryptosporidium*
	Bleach	*Encephalitozoon*, *Dipylidium*
	Cresol	*Cystoisospora*, *Cryptosporidium*, *Giardia*
	Hydrogen peroxide	*Cryptosporidium*, *Giardia*,
	Iodine	*Ancylostoma*,
	Ozone	*Giardia*
	Quaternary ammonium	*Leishmania*, *Toxoplasma*, *Giardia*

## Data Availability

Not applicable.
